# CRPS Is Not Associated with Altered Sensorimotor Cortex GABA or Glutamate

**DOI:** 10.1523/ENEURO.0389-19.2020

**Published:** 2020-02-14

**Authors:** Barbara Lee, Luke A. Henderson, Caroline D. Rae, Flavia Di Pietro

**Affiliations:** 1 Department of Anatomy and Histology, Brain and Mind Centre, University of Sydney, Sydney 2006, Australia; 2 Neuroscience Research Australia, Sydney 2031, Australia; 3 School of Pharmacy and Biomedical Sciences, Faculty of Health Sciences, Curtin University, Perth 6102, Australia

**Keywords:** chronic pain, cortical reorganization, GABA, glutamate, inhibition, sensorimotor cortex

## Abstract

Complex regional pain syndrome (CRPS) is a debilitating chronic pain disorder typically in the upper or lower limbs. While CRPS usually develops from a peripheral event, it is likely maintained by CNS changes. Indeed, CRPS is reported to be associated with sensorimotor cortex changes, or functional “reorganization,” as well as deficits such as poor tactile acuity. While the mechanisms underpinning cortical reorganization in CRPS are unknown, some have hypothesized that it involves disinhibition (i.e., a reduction in GABA activity). In this study, we addressed this hypothesis by using edited magnetic resonance spectroscopy to determine sensorimotor GABA and glutamate concentrations in 16 humans with CRPS and 30 matched control subjects and the relationship of these concentrations with tactile acuity. We found that individuals with upper limb CRPS displayed reduced tactile acuity in the painful hand, compared with the nonpainful hand and pain-free control subjects. Despite this acuity deficit, CRPS was not associated with altered GABA or glutamate concentrations within the sensorimotor cortex on either the side that represents the affected or unaffected hand. Furthermore, there was no significant relationship between sensorimotor GABA or glutamate concentrations and tactile acuity in CRPS subjects or control subjects. Although our sample was small, these data suggest that CRPS is not associated with altered total sensorimotor GABA or glutamate concentrations. While these results are at odds with the sensorimotor cortex disinhibition hypothesis, it is possible that GABAergic mechanisms other than total GABA concentration may contribute to such disinhibition.

## Significance Statement

Complex regional pain syndrome is a debilitating chronic pain disorder that usually affects the limbs. It is associated with altered sensorimotor cortex function including reorganization and reduced tactile acuity, which are thought to result from reduced ongoing inhibition. However, we found that this pain condition is not associated with reduced ongoing sensorimotor inhibition in the form of GABA concentration, the major inhibitory neurotransmitter in the brain. These findings strongly suggest that changes in sensorimotor function in individuals with complex regional pain syndrome are explained by factors other than neurotransmitter concentration.

## Introduction

Complex regional pain syndrome (CRPS) is a debilitating chronic pain disorder characterized by spontaneous or regionally evoked pain lasting for more than 3 months that typically affects the distal extremities, particularly the upper limbs (Marinus et al., 2011). CRPS often develops following an injury such as a fracture, but it can develop spontaneously (Marinus et al., 2011). While CRPS usually develops from a peripheral event, it is thought to be maintained by changes in the CNS since it often spreads to other body regions in a nondermatome fashion (van Rijn et al., 2011). There is no relationship between the severity of an injury and the development of CRPS; however, fracture patients who perceive excessive pain [visual analog scale (VAS) score, ≥5 of 10] in the first week after fracture are more likely to develop CRPS than patients who report lower pain intensity (Moseley et al., 2014). Further evidence of CNS involvement is the finding that CRPS patients often exhibit perceptual deficits such as a neglect-like syndrome (Galer and Jensen, 1999), distorted mental perceptions of their affected limbs (Moseley, 2005), and frequent mislocalized tactile perceptions and referred sensations (McCabe et al., 2003). Despite evidence of higher neural involvement, the underlying mechanisms responsible for the development of CRPS remain unknown, and there is no definitive treatment or cure (Marinus et al., 2011).

There is growing evidence that CRPS is associated with changes in primary sensorimotor (S1/M1) cortex function. Functional neuroimaging investigations have demonstrated that CRPS patients display primary somatosensory cortex (S1) “reorganization,” whereby the S1 region responsible for the CRPS-affected hand is smaller than that of the unaffected hand (Juottonen et al., 2002; Maihöfner et al., 2003, 2004; Pleger et al., 2004; Vartiainen et al., 2009; Di Pietro et al., 2013a). This is thought to underlie the perceptual abnormalities seen in these patients; the most commonly reported is tactile acuity via two-point discrimination testing (TPD), which is simply the smallest distance between two points that is correctly perceived as two points, rather than one, touching the skin. CRPS patients have poor tactile acuity on their affected limbs (i.e., a high TPD threshold), and this has been associated with the degree of cortical reorganization (Pleger et al., 2006; Maihöfner and DeCol, 2007; Peltz et al., 2011; Reiswich et al., 2012; Catley et al., 2014; David et al., 2015). The primary motor cortex (M1) is also altered in CRPS, with reported changes in M1 function and excitability in CRPS patients (Di Pietro et al., 2013b). Although the fundamental mechanisms underpinning cortical reorganization in CRPS are unknown, some have suggested that a change in ongoing inhibition, particularly disinhibition, may underlie the S1/M1 changes in CRPS. Indeed, both S1 and M1 disinhibition have been detected in CRPS (Schwenkreis et al., 2003; Eisenberg et al., 2005; Lenz et al., 2011; Di Pietro et al., 2013a,b). Evidence suggests that S1 and M1 exhibit reduced intracortical inhibition compared with healthy control subjects, hence CRPS patients have been proposed to have bilateral disinhibition of the sensorimotor cortex. While studies postulate that the disinhibition in CRPS is associated with changes in GABA activity, no study has investigated GABA concentration in the sensorimotor cortex of CRPS patients or the balance between GABA and glutamate. This is surprising and somewhat of an oversight given that there are treatments that have been developed and adopted clinically that are theoretically aimed at restoring CNS inhibition (Moseley et al., 2012).

The aim of this study was to use magnetic resonance spectroscopy (MRS) to investigate sensorimotor GABA concentration in CRPS subjects. We hypothesized that sensorimotor GABA concentration in CRPS subjects would be decreased compared with healthy control subjects. Furthermore, since GABA and glutamate work together to maintain excitatory/inhibitory balance, we also investigated sensorimotor glutamate concentration and hypothesized that it would remain at control levels in CRPS. Finally, since greater sensorimotor GABA concentration has been found to predict better tactile acuity performance (Puts et al., 2011; Kolasinski et al., 2017) and individuals with CRPS have poor tactile acuity (Catley et al., 2014), we hypothesized that CRPS subjects would have reduced tactile acuity and that this would be correlated with reduced sensorimotor GABA concentration.

## Materials and Methods

Because of the challenging nature of recruiting an eligible and willing sample of participants with upper limb CRPS, no sample size calculation was performed; the study recruited a convenience sample. Sixteen subjects with CRPS (12 females; mean ± SEM age, 48 ± 3 years) and 30 pain-free healthy control subjects (17 females; mean ± SEM age, 34 ± 2 years) were recruited for the study. CRPS subjects were recruited and their condition diagnosed in accordance with the International Association for the Study of Pain “Budapest” diagnostic criteria (Harden et al., 2007) and had ongoing pain for at least 3 months. For each subject, handedness was assessed using the Edinburgh Handedness Inventory (Oldfield, 1971). Subjects were excluded if they did not meet standard MRI safety criteria and healthy control subjects were excluded if they experienced any chronic pain condition. Informed written consent was obtained for all procedures, which were conducted under the approval by local Institutional Human Research Ethics Committees and consistent with the Declaration of Helsinki.

For the CRPS subjects, sensory signs of hyperalgesia and allodynia were assessed via pinprick on the dorsal web space of the hand and light brush strokes on the dorsum of the hands/forearms respectively. Vasomotor signs of skin temperature asymmetry were assessed through touch, skin color changes/asymmetry through visual observation and sudomotor/edema signs of sweating via touch, and edema was determined using a tape measure. Motor signs were assessed by visual observation of finger, hand, and wrist movement. Trophic changes to hair, nail, and skin were visually assessed. CRPS subjects assessed the intensity of their ongoing pain using a VAS (0 = no pain to 10 = worst pain imaginable) three times per day for 7 consecutive days during the week of the scanning session. The average of these pain ratings was taken as a measure of “diary pain” intensity. Subjects also described their pain distribution by outlining the area of their chronic pain on a standard drawing of the body and assessed their pain on the day of the scanning session on a 10 cm VAS (i.e., “scan pain” intensity).

### Tactile acuity

**Figure 1.**
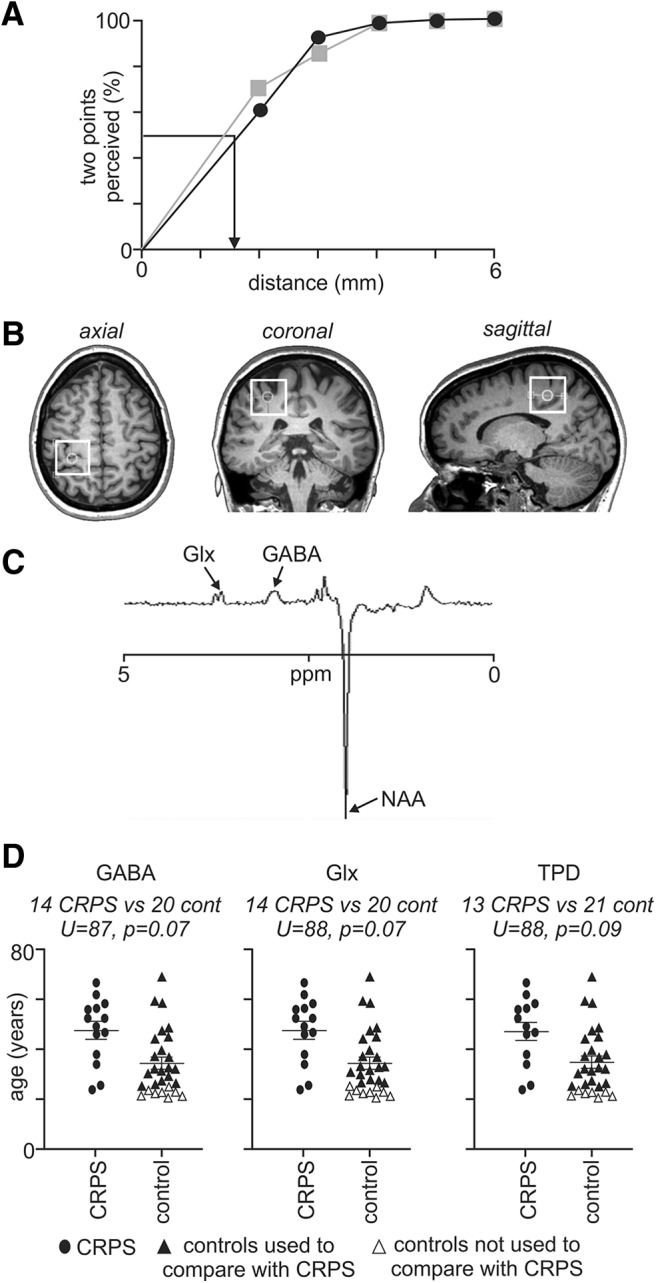
***A***, A psychometric function of two-point discrimination threshold for a single subject’s index finger. The percentage of two-point perception was plotted against the different point-to-point distances tested in the two-point discrimination task. The gray curve with gray squares indicates the subject’s percentage of correct responses for each distance, and the black curve with circles is the fitted binary logistic regression. The two-point discrimination threshold is the distance at which correct two-point perception was at 50% (black arrow). ***B***, MRS voxel placement on the sensorimotor cortex. Voxel placement over the left sensorimotor cortex of a single subject indicated by the white square outline. The voxel was guided by the “hand hook” in the sagittal plane and the "hand knob" in the axial plane. ***C***, A typical MEGA-PRESS spectrum obtained from the sensorimotor cortex. NAA, *N*-acetyl aspartate. ***D***, Plots demonstrating the age distribution of the two groups. The left plot shows subjects used for GABA, the middle plot for Glx, and the right plot for TPD. While there were significant differences between groups if all control and CRPS subjects were used for the analyses, restriction of the control group numbers allowed for group comparisons in which there was no overall significant difference in age (Mann–Whitney *U* test, *p* > 0.05), sex (χ^2^ test, *p* > 0.05), or handedness (Mann–Whitney *U* test, *p* > 0.05).

Tactile acuity was measured with a TPD assessment. TPD was assessed following the MRS scan to prevent possible influences of tactile acuity training on neurochemistry (Heba et al., 2016; Schmid et al., 2017). Subjects were instructed to rest their hand in a supine position and to keep their eyes closed, with the order of testing randomized. A TPD wheel (Exacta) was applied longitudinal to the surface of the distal pulp of the index finger until the first skin blanching around the points. Each distance was presented seven times in a randomized order, resulting in a total of 35 trials. The subjects reported whether they felt one point or two points touching the skin. When the subject could not feel two points at 5 mm spacing, then the distance was gradually increased until two points could be felt. The percentage of two-point perception was plotted against the distance between the points and fitted by a binary logistic regression (SPSS Statistics for Windows, version 24.0, IBM), resulting in a psychometric function of absolute threshold. From the binary logistic regression fit, the threshold was determined as the distance at which the chance level (50%) of correct two-point perception was reached (Fig. 1*A*).

### MRI scans

Each subject was placed in the supine position on the MRI scanner bed in a 3 Tesla MRI scanner (Achieva TX, Philips Medical Systems), with their head in a 32-channel head coil to which padding was added to prevent head movement. With the subject relaxed, a high-resolution T1-weighted anatomical image of the whole brain was collected (288 axial slices, repetition time = 5600 ms; raw voxel size = 0.87 × 0.87 × 0.87 mm thick). Using this T1-weighted anatomical image set, a 30 × 30 × 30 mm voxel was placed over the right and then separately over the left sensorimotor cortex over the region that represents the hand (Fig. 1*B*). All three planes were referenced to ensure that the voxel position minimized the inclusion of cerebrospinal fluid (CSF), did not incorporate dura mater, and did not enter the ventricles.

The GABA-edited Mescher-Garwood Point Resolved Spectroscopy (MEGA-PRESS) method of MRS was then performed on the 30 × 30 × 30 mm voxel, on the left and right sides individually (repetition time = 1800 ms; echo time = 68 ms; 2048 data points, acquisition time = ∼8 min). The GABA resonance at 3.01 ppm was acquired by applying the MEGA-PRESS edited pulse at 1.9 ppm (“ON” spectra) and at 7.46 ppm (“OFF”) in interleaved scans.

### MRS analysis

Because of the similar chemical structure of glutamate and glutamine, the separation of glutamate and glutamine is problematic at 3 T field strength (Govindaraju et al., 2000). The combination of glutamate and glutamine is referred to as Glx; Glx and glutamate are used interchangeably here. The Glx peak in edited MEGA-PRESS spectra is largely proportional to the concentrations of glutamate and glutamine (Shungu et al., 2013), and, as glutamate concentrations are generally reported to be approximately threefold to fourfold higher than glutamine, the Glx peak can be considered to be derived proportionately more from glutamate than from glutamine (Rae, 2014), although relative changes in either metabolite cannot reliably be determined from the Glx resonance (Sanaei Nezhad et al., 2017). All MRS data were processed using the Java-based Magnetic Resonance User’s Interface version 3 (jMRUI 3.0, MRUI Consortium). The ON and OFF spectral subsets were separately summed to produce single ON and OFF subspectra for each spectral dataset. The ON and OFF subspectra were then subtracted, resulting in GABA-edited difference spectra to measure GABA concentration at 3.01 ppm and Glx concentration at 3.75 ppm (Fig. 1*C*). The dominant water resonance was removed from the difference spectra using the Hankel–Lanczos Singular Values Decomposition Filter tool. GABA and Glx concentrations were quantified using the Advanced Method for Accurate, Robust and Efficient Spectral fitting (AMARES), a nonlinear least-squares fitting algorithm operating in the time domain. Peak fitting for GABA and Glx was performed after manually defining the center frequency and line width of GABA and Glx peaks. Lorentzian curves were used to obtain the peak amplitudes for GABA and Glx.

The OFF spectra subsets were summed to produce a single OFF subspectra to measure creatine (Cr) concentration at 3.02 ppm. Cr concentration was quantified using QUEST (QUantification ESTimation), a time-domain algorithm that fits a weighted metabolite basis set to the spectra acquired (Ratiney et al., 2005). GABA and Glx concentrations were expressed relative to creatine (i.e., GABA/Cr and Glx/Cr ratios). To ensure that there were no tissue fraction differences between hemispheres and groups (e.g., CRPS vs control subjects), the fraction of gray matter (GM), white matter (WM) and CSF was measured for each MEGA-PRESS 30 × 30 × 30 mm voxel. The GM, WM, and CSF fractions were acquired using MATLAB (MathWorks) and the partial volume code obtained from the Bangor Imaging Unit website (Gasparovic et al., 2006; Goulden and Mullins, 2020). The signal-to-noise ratio was defined in the frequency domain as the maximum height of the largest metabolite peak divided by the root mean square (rms) amplitude of noise in a signal-free and artifact-free part of the spectrum. The mean (±SEM) signal-to-noise ratio for GABA was 0.47 ± 0.01 (range, 0.29–0.64), and for Glx was 1.34 ± 0.11 (range, 0.18–2.72).

### Statistical analysis

All statistical analyses were performed using GraphPad Prism 7.0 (GraphPad Software). All data were first tested for normality via the D’Agostino and Pearson normality test. For normally distributed data, parametric tests were used; paired *t* tests were used for within-subject comparisons and unpaired *t* tests were used for between-group comparisons. Nonparametric tests were used for non-normally distributed data; Wilcoxon matched-pairs signed rank test (W) for within-subject and Mann–Whitney *U* test (U) for between-group comparisons. To account for GM and WM differences, SPSS Statistics (version 24.0, IBM) was used to run ANCOVA for MRS GABA and Glx comparisons with GM/WM ratios as covariates. There was no difference in significance with GM and WM as covariates (not reported here).

## Results

Two of the 16 CRPS subjects were excluded from the MRS analysis, and 2 of the control subjects were excluded from the GABA and 1 from the glutamate analysis due to technical difficulties and claustrophobia. The demographics and clinical characteristics of the remaining 14 CRPS subjects are shown in Table 1. All 14 CRPS subjects reported ongoing pain in the upper limb and 7 also reported pain in the lower limb. Eleven of the 14 subjects reported pain in the upper limb restricted to one side of the body, 9 on the right and 2 on the left, and the remaining 3 reported bilateral pain, although pain was greater on the right side in 2 and on the left in 1 of these subjects. For CRPS subjects, the S1/M1 contralateral to the greatest ongoing upper limb pain was considered to be the “affected” side and vice versa for the “unaffected” side (i.e., there were 11 right-sided pain and 3 left-sided pain).

**Table 1:** Demographics and clinical characteristics of patients with CRPS

	** **	** **	** **	** **	** **	** **	** **	** **	Signs (symptoms)				** **	** **
Subject	Age	Sex	EHI score (handedness)	Pain duration (years)	CRPS affected region	Inciting event	Medications	Comorbidity	Sensory	Vaso-motor	Sudomotor/edema	Motor/trophic	Pain intensity (diary VAS)	Pain intensity (day VAS)
1	49	M	100.0 (R)	7.0	**R UL**, R LL, L LL, face, abdomen	Pain in R hand	Turmeric tablets	None	+ (+)	+ (+)	+ (+)	+ (+)	4.5	4.0
2	56	F	100.0 (R)	4.2	**L UL**, R UL, R LL, R and L chest	L humerus fracture	Duloxetine, gabapentin, oxycodone, quetiapine, tapentadol	L radial nerve palsy, Triangular fibrocartilage complex of R hand	+ (+)	+ (+)	+ (+)	+ (+)	8.1	7.8
3	56	F	60.0 (R)	0.9	**R UL**, R neck, R chest	Spontaneous onset	Ashwagandha, budesonide, cannabis, codeine, formoterol, oxycodone, paracetamol, salbutamol	Back pain, COPD, fibromyalgia, osteoarthritis, peptic ulcer, radiculopathy, Raynaud’s disease, spinal disc herniation	+ (+)	+ (+)	− (+)	+ (+)	8.3	7.9
4	62	F	100.0 (R)	6.2	**L UL**, *R UL* ^2^	R hand tendon release surgery	Amitriptyline, cannabidiol drops, codeine **,** levothyroxine, magnesium, paracetamol, topiramate, tramadol, valerian	Diverticulitis, gastroesophageal reflux disease, Graves’ disease (thyroidectomized)	+ (+)	+ (+)	− (+)	+ (+)	5.8	4.3
5	58	F	−20.0 (A)	8.7	**R UL**, R LL, R face	R arm surgery	Codeine, duloxetine, linagliptin, meloxicam, metformin, paracetamol	Diabetes	+ (+)	+ (+)	+ (+)	+ (+)	4.7	4.1
6	67	F	100.0 (R)	9.5	**R UL**, L UL, R LL, L LL	R radius fracture	Amlodipine, gabapentin, ketamine in lipoderm cream, metformin, metoprolol, pantoprazole, salbutamol	Asthma, diabetes, gastric reflux, hypertension, osteoarthritis, pubic symphysitis, supraventricular tachycardia	+ (+)	− (+)	− (+)	+ (+)	3.7	5.0
7	47	M	44.4 (R)	1.5	**R UL**	Spontaneous onset	Amlodipine, atorvastatin, ibuprofen, paracetamol, perindopril, pregabalin	Hyperlipidemia, hypertension	+ (+)	+ (+)	− (−)	+ (+)	6.8	7.1
8	34	F	−23.1 (A)	5.3	**R UL**, R LL, R hip	R wrist fracture	Amitriptyline, buprenorphine patch	Migraine, R hip bursitis	+ (+)	+ (+)	+ (+)	+ (+)	4.3	2.4
9	26	F	−40.0 (A)	1.3	**R UL**, L and R neck, spine, L LL	R hand nerve damage	None	Endometriosis, polycystic ovarian syndrome	+ (+)	+ (+)	+ (+)	+ (+)	5.4	6.8
10	46	F	80.0 (R)	3.9	**L UL**	L hand carpal tunnel release surgery	Amitriptyline, betahistine, duloxetine, naproxen, pantoprazole, rizatriptan, tapentadol, valaciclovir	Carpal tunnel of R hand, fibromyalgia, herpes, migraine, polycystic ovarian syndrome with insulin resistance, vertigo	+ (−)	+ (+)	+ (+)	− (+)	0.6	5.6
11	24	F	70.0 (R)	2.6	**R UL**, L UL	Overload	Amitriptyline, gabapentin, levothyroxine	Hashimoto’s disease	− (−)	− (+)	+ (+)	+ (+)	4.5	4.4
12	52	F	40.0 (A)	2.9	**R UL**, R torso	Broke tailbone	Ashwagandha, fish oil, ibuprofen, magnesium, mega B, melatonin, paracetamol, tapentadol, vitamin C, vitamin D	Endometriosis	+ (+)	+ (+)	− (+)	− (+)	3.8	3.5
13	38	F	17.6 (A)	12.7	**R UL**, R neck, L LL	Spontaneous onset	Duloxetine, gabapentin, naloxone, oxycodone, palmitoylethanolamide (PEA)	Endometriosis, endosalpingiosis, Raynaud’s disease	+ (+)	+ (+)	+ (+)	+ (+)	0.0	5.9
14	52	M	−88.9 (L)	1.9	**R UL**, L and R neck, back	R scaphoid fusion surgery	Cholecalciferol, ibuprofen, magnesium, oxycodone, paracetamol, pregabalin, tramadol, venlafaxine, zopiclone	L shoulder bursitis, Sleep apnea	+ (+)	+ (+)	+ (+)	+ (+)	7.0	7.6

R, Right; L, left; A, ambidextrous; UL, upper limb; LL, lower limb; +, present; −, absent. Bold type indicates the CRPS region with the most severe pain. Italic type indicates remission of the CRPS region. Underline indicates medication taken in the last 24 h of the day of testing. The presence (+) or absence (-) of CRPS signs and symptoms are presented as signs (symptoms).

### GABA

**Figure 2.**
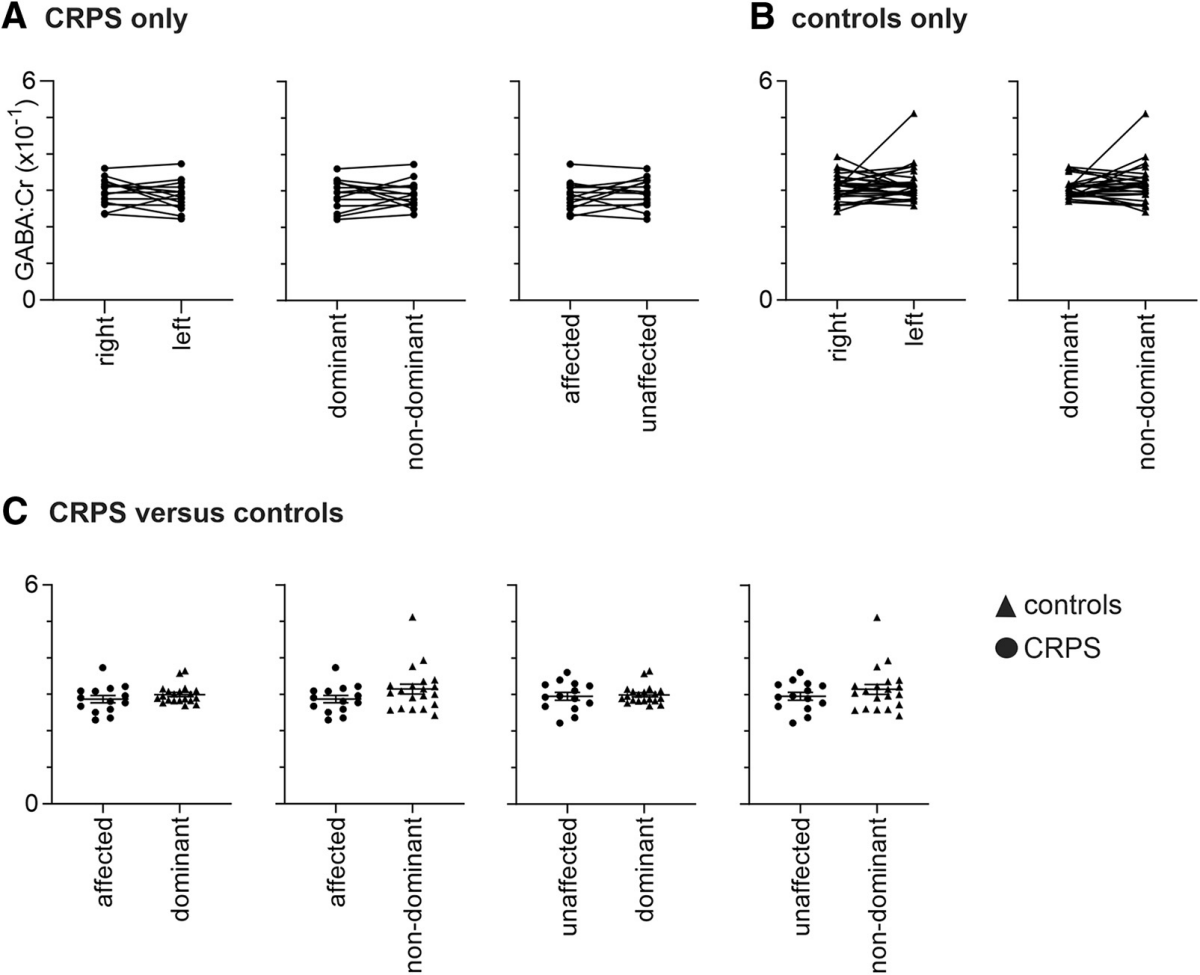
Plots of GABA/Cr ratios (×10^−1^) of a 30 × 30 × 30 mm voxel placed over the hand representation of the sensorimotor cortex. ***A***, Values for individual subjects with upper limb CRPS for the right and left, dominant and nondominant hemispheres and also for the affected (hemisphere representing the side of ongoing pain) and unaffected hemispheres. Plots show pairwise connections for each individual subject. ***B***, Values for individual control subjects. ***C***, Values comparing individual CRPS and control subjects. Horizontal lines indicate the mean ± SEM for each group. Note there are no significant differences between any hemisphere in the CRPS or control subjects alone or between CRPS and control groups.

In the 11 subjects with unilateral pain, there was no difference in GABA concentration between the hemispheres representing the painful (affected) and nonpainful (unaffected) limbs (mean ± SEM ×10^−1^ GABA/Cr ratio: affected, 2.82 ± 0.12; unaffected, 2.91 ± 0.13; *p* = 0.49). Similarly, for the whole group of 14 CRPS subjects, we found no significant differences in S1/M1 GABA between the right and left hemispheres (right, 2.86 ± 0.11; left, 2.96 ± 0.10; *p* = 0.32) or between the dominant (handedness) and nondominant hemispheres (dominant, 2.91 ± 0.11; nondominant, 2.92 ± 0.11; *p* = 0.94; Fig. 2*A*). For the 28 control subjects, we also found no significant differences between the right and left hemispheres (right, 3.07 ± 0.06; left, 3.17 ± 0.09; *p* = 0.51) or between the dominant and nondominant hemispheres (dominant, 3.06 ± 0.05; nondominant, 3.18 ± 0.09; *p* = 0.36; Fig. 2*B*).

To address the primary aim of characterizing GABA concentration in CRPS, a group of 20 control subjects was selected such that there was no significant difference between control subjects and CRPS subjects with respect to age (Fig. 1*D*; Mann–Whitney *U* test, *p* > 0.05), sex (χ^2^ test, *p* > 0.05) and handedness (Mann–Whitney *U* test, *p* > 0.05). We found no significant difference in S1/M1 GABA when comparing the CRPS-affected with control subjects’ dominant (affected, 2.87 ± 0.10; dominant, 2.95 ± 0.11; *p* = 0.36), CRPS-affected versus control subjects’ nondominant (affected, 2.87 ± 0.10; nondominant, 3.14 ± 0.14; *p* = 0.15), CRPS-unaffected versus control subjects’ dominant (unaffected, 2.95 ± 0.11; dominant, 2.95 ± 0.11; *p* = 0.99), or CRPS-unaffected versus control subjects’ nondominant (unaffected, 2.95 ± 0.11; nondominant, 3.14 ± 0.14; *p* = 0.64; Fig. 2*C*).

**Figure 3.**
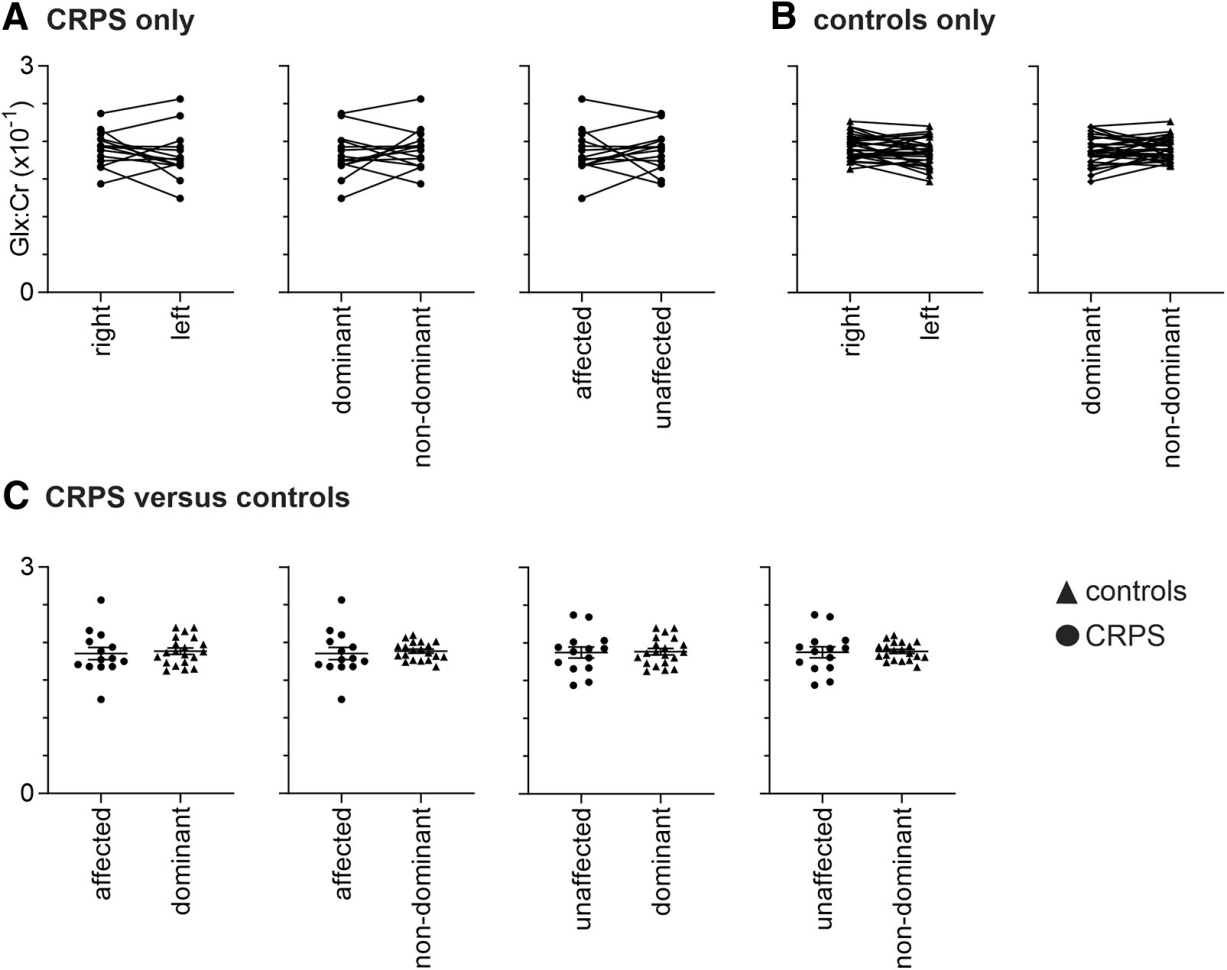
Plots of Glx/Cr ratios (×10^−1^) of a 30 × 30 × 30mm voxel placed over the hand representation of the sensorimotor cortex. ***A***, Values for individual subjects with upper limb CRPS for the right and left hands, dominant and nondominant hemispheres, and also for the affected (hemisphere representing the side of ongoing pain) and unaffected hemispheres. Plots show pairwise connections for each individual subject. ***B***, Values for individual control subjects. ***C***, Values comparing individual CRPS subject and control subject groups. Horizontal lines indicate the mean ± SEM for each group. Note that there are no significant differences between any hemisphere in the CRPS or control subjects alone or between CRPS and control groups.

### Glx

Similar to the findings for GABA, in the 11 subjects with unilateral pain, we found no difference between the hemispheres representing the affected and unaffected limbs (mean ± SEM × 10^−1^ Glx/Cr ratio: affected, 1.88 ± 0.10; unaffected, 1.90 ± 0.08; *p* = 0.79; Fig. 3*A*). In the group of 14 CRPS subjects, we found no significant differences in S1/M1 Glx between the right and left hemispheres (right, 1.90 ± 0.06; left, 1.83 ± 0.08; *p* = 0.30) or between the dominant and nondominant hemispheres (dominant, 1.83 ± 0.08; nondominant, 1.89 ± 0.07; *p* = 0.45). For the 29 control subjects, we also found no significant differences between the right and left hemispheres (right, 1.93 ± 0.03; left, 1.86 ± 0.03; *p* = 0.07) or between the dominant and nondominant hemispheres (dominant, 1.89 ± 0.04; nondominant, 1.90 ± 0.03; *p* = 0.65; Fig. 3*B*).

To determine the differences between CRPS and control subjects, we analyzed all 14 CRPS subjects against a group of 20 control subjects so that there was no significant difference between control subjects and CRPS subjects with respect to age (Fig. 1*D*; Mann–Whitney *U* test, *p* > 0.05), sex (χ^2^ test, *p* > 0.05), and handedness (Mann–Whitney *U* test, *p* > 0.05). We found no significant difference in S1/M1 Glx when comparing the CRPS-affected subjects to control subjects’ dominant (affected, 1.85 ± 0.08; dominant, 1.89 ± 0.04; *p* = 0.72), the CRPS-affected subjects to control subjects’ nondominant (affected, 1.85 ± 0.08; nondominant, 1.88 ± 0.03; *p* = 0.69), the CRPS-unaffected subjects to control subjects’ dominant (unaffected, 1.87 ± 0.07; dominant, 1.89 ± 0.04; *p* = 0.88), or the CRPS-unaffected subjects to control subjects’ nondominant (unaffected, 1.87 ± 0.07; nondominant, 1.88 ± 0.03; *p* = 0.87; Fig. 3*C*).

### Tactile acuity

**Figure 4.**
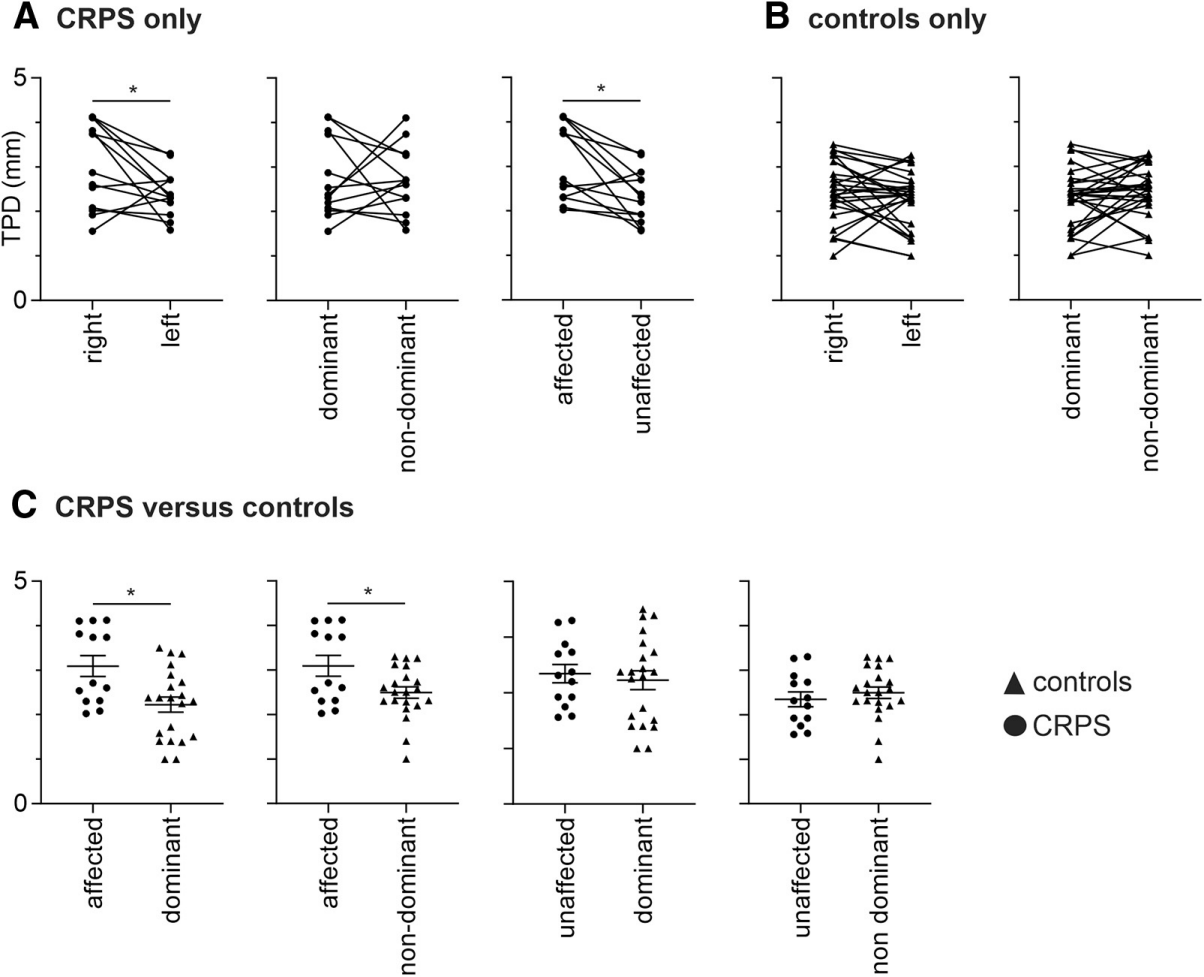
Plots of TPD in millimeters. ***A***, Values for individual subjects with upper limb CRPS for the right and left hands, dominant and nondominant hands, and also for the affected (hand with ongoing pain) and unaffected hands. Plots show pairwise connections for each individual subject. ***B***, Values for individual control subjects. ***C***, Values comparing individual CRPS subject and control subject groups. Horizontal lines indicate the mean ± SEM for each group. Note that CRPS subjects displayed reduced tactile acuity (increased TPD) in the affected hand compared with the unaffected hand and the dominant and nondominant hands in control subjects (*p < 0.05).

Three of the 16 CRPS subjects were excluded from TPD analysis due to ongoing pain preventing testing, and 2 of the 30 control subjects due to the perception of two points during one-point stimuli runs. As hypothesized, in CRPS patients, the affected hand had a significantly greater TPD threshold (i.e., reduced tactile acuity) than the less/unaffected hand (mean ± SEM TPD in millimeters; affected, 3.09 ± 0.23; unaffected, 2.35 ± 0.16; *p* = 0.004), and, given that most CRPS subjects had right side pain in the upper limb, there was a significant TPD threshold difference between the right and left hands (right, 3.02 ± 0.25; left, 2.41 ± 0.14; *p* = 0.04), but no difference between the dominant and nondominant hands (dominant, 2.74 ± 0.24; nondominant, 2.69 ± 0.20; *p* = 0.87; Fig. 4*A*). In control subjects, we found no significant difference between the right and left hands (right, 2.45 ± 0.13; left, 2.27 ± 0.12; *p* = 0.11) or the dominant and nondominant hands (dominant, 2.26 ± 0.13; nondominant, 2.45 ± 0.11; *p* = 0.08; Fig. 4*B*).

To determine the differences between CRPS and control subjects, we analyzed all 13 CRPS subjects against a group of 21 control subjects so that there was no significant difference between control subjects and CRPS subjects with respect to age (Fig. 1*D*; Mann–Whitney *U* test, *p* > 0.05), sex (χ^2^ test, *p* > 0.05), and handedness (Mann–Whitney *U* test, *p* > 0.05). This comparison revealed that the CRPS-affected hand had significantly greater TPD threshold, and hence poorer tactile acuity, than the dominant hand of the control subjects (affected, 3.09 ± 0.23; dominant, 2.23 ± 0.17; *p* = 0.007) and the nondominant hand of the control subjects (affected, 3.09 ± 0.23; nondominant, 2.49 ± 0.13; *p* = 0.04; Fig. 4*C*). However, tactile acuity for the CRPS-unaffected hand was not different from the dominant hand of the control subjects (unaffected, 2.35 ± 0.16; dominant, 2.23 ± 0.17; *p* = 0.32) or the nondominant hand of control subjects (unaffected, 2.35 ± 0.16; nondominant, 2.49 ± 0.13; *p* = 0.24).

Although we found a significant difference in TPD threshold in CRPS subjects between the affected and unaffected hands, there were no significant correlations between TPD in the affected hand and GABA or Glx in the affected S1/M1 (GABA: *r* = 0.49, *p* = 0.06; *r* = 0.17, *p* = 0.59), or between TPD in the unaffected hand and GABA or Glx in the unaffected S1/M1 (GABA: *r* = 0.12, *p* = 0.35; Glx: *r* = −0.09, *p* = 0.77). Similarly in control subjects, there were no significant correlations between TPD in the dominant hand and GABA or Glx in the dominant S1/M1 (GABA: *r* = 0.09, *p* = 0.32; Glx: *r* = 0.24, *p* = 0.12) or between TPD in the nondominant hand and GABA or Glx in the nondominant S1/M1 (GABA: *r* = 0.24, *p* = 0.12; Glx: *r* = 0.01, *p* = 0.99).

## Discussion

Contrary to our hypothesis, we found no significant difference in primary sensorimotor cortex GABA concentration in CRPS subjects compared with control subjects. Furthermore, although the affected hand displayed poor tactile acuity relative to the unaffected hand, there was no difference in GABA concentration in the S1/M1 region representing the affected compared with the unaffected hand in CRPS subjects. As hypothesized, we found no significant difference in sensorimotor cortex glutamate concentration in CRPS subjects compared with control subjects or in CRPS subjects between the affected and unaffected hemispheres. For the first time in the field, these findings suggest that ongoing pain and reduced tactile acuity in CRPS subjects may not result from altered GABA or glutamate concentrations in the primary sensorimotor cortex.

Transcranial magnetic stimulation (TMS) studies have identified reduced short intracortical inhibition in the sensorimotor cortex in individuals with CRPS, and it has been postulated that this sensorimotor disinhibition results from reduced sensorimotor cortex GABAergic inhibition (Schwenkreis et al., 2003; Eisenberg et al., 2005; Lotze and Moseley, 2007; Lenz et al., 2011). While Lenz et al. (2011) and Schwenkreis et al. (2003) reported bilateral sensorimotor cortex disinhibition, Eisenberg et al. (2005) reported more disinhibition in the sensorimotor cortex representing the affected limb than the unaffected one. In any case, we found no difference in GABA concentration in CRPS subjects relative to control subjects or between the hemispheres representing the affected limb compared with the unaffected limb. Furthermore, although we found that CRPS subjects displayed reduced tactile acuity in the affected hand, this altered acuity was not associated with altered contralateral sensorimotor GABA concentration. Consistent with this lack of difference, we also found no difference in sensorimotor glutamate concentrations and no relationship to tactile acuity in control subjects or CRPS subjects. While the lack of difference in GABA is surprising, the lack of change in glutamate is not. It has been reported that glutamate activity is positively correlated with intracortical facilitation (Liepert et al., 1997) and cortical silent period duration (Tremblay et al., 2013) in healthy control subjects. Given that in CRPS patients, intracortical facilitation and cortical silent period durations are not different from healthy control subjects (Schwenkreis et al., 2003; Krause et al., 2005), as we hypothesized, sensorimotor glutamate concentration was not different between CRPS subjects and healthy control subjects.

It is possible that a reduction in inhibition is not reflected in “ongoing” levels of GABA. A recent investigation found that TMS physiological disinhibition was not significantly correlated to MRS measures of sensorimotor GABA concentration (Dyke et al., 2017), and it has been proposed that TMS-related inhibition may better reflect transient phasic GABAergic signaling dependent on GABA_A_ receptor activity (Stagg et al., 2011a). Indeed, it has been previously proposed that MRS is a measure of tonic inhibition and that the GABA concentration measured by MRS is largely extrasynaptic GABA, which better reflects the amount of GABA that can be released for tonic inhibition (Nasrallah et al., 2011; Ziemann, 2013; Rae, 2014; Stagg, 2014). Hence, it is possible that the disinhibition in CRPS examined using TMS reflects phasic disinhibition and not tonic inhibition as measured by MRS in this study. Consistent with this idea, experimental animal investigations have shown that blocking GABA_A_ receptor activity results in an expansion of the size of cortical representation within S1 and M1 (Tremere et al., 2001; Chowdhury and Rasmusson, 2002; Capaday and Rasmusson, 2003), and the short intracortical inhibition revealed using TMS in CRPS may reflect reduced activation of synaptic GABA_A_ receptors on GABAergic interneurons (Eisenberg et al., 2005; Ziemann et al., 2015).

It is generally accepted that CRPS is associated with S1 reorganization, with the hemisphere receiving input from the painful limb displaying a smaller functional representation (Juottonen et al., 2002; Maihöfner et al., 2003, 2004; Pleger et al., 2004; Vartiainen et al., 2009; Di Pietro et al., 2013a). In addition, in a recent investigation of upper limb CRPS, it was reported that, as well as reduced S1 representation of the affected hand, there was a significant expansion of the unaffected hand representation compared with control subjects (Di Pietro et al., 2015). Interestingly, this expansion of the healthy hand representation did not appear to relate to overall hand use or the severity of dysfunction of the painful hand (Di Pietro et al., 2016). Given these findings, it is difficult to reconcile how bilateral short intracortical inhibitory changes in CRPS and the proposed role for GABA_A_ in cortical organization relate to such opposing changes in S1 cortical organization in the affected compared with the unaffected hemispheres. It is possible that disinhibition in CRPS may be associated with differences in synaptic GABA_A_ receptor activity rather than total GABA concentration, but how this is related to S1 organization remains unknown.

Although it is possible that differences in synaptic GABA_A_ receptor activity may account for TMS-associated phasic disinhibition in CRPS, it is noted that extrasynaptic GABA, when released by neural activation (Nasrallah et al., 2011) can act on extrasynaptic GABA_A_ receptors, which are thought to mediate more tonic changes in activity (Semyanov et al., 2003; Yeung et al., 2003; Farrant and Nusser, 2005). MRS measures total GABA concentration and does not separate GABA into functional pools (e.g., extracellular, vesicular, cytoplasmic; Stagg et al., 2011a). Thus, while total GABA concentration of CRPS patients may not differ from healthy control subjects, the ratio of GABA in each pool may differ. Increased extracellular GABA can increase the tonic inhibition of neurons, making neurons less likely to activate and potentially resulting in disinhibition, and thus it is possible that both synaptic GABA_A_ receptor activity and extracellular GABA concentration contribute to disinhibition in CRPS (Farrant and Nusser, 2005).

As hypothesized and consistent with previous literature, the CRPS-affected hand displayed poor tactile acuity relative to the CRPS-unaffected hand and pain-free control subjects (Pleger et al., 2006; Maihöfner and DeCol, 2007; Peltz et al., 2011; Lewis and Schweinhardt, 2012; Reiswich et al., 2012; Catley et al., 2014; David et al., 2015). While in healthy control subjects, one might hypothesize that increased use of the dominant hand would result in better tactile acuity than the nondominant hand, we found no difference in dominant and nondominant tactile acuity in control subjects, a result consistent with findings of previous investigations (Gelber et al., 1995; Ozcan et al., 2004; Pleger et al., 2006; Tustumi et al., 2015). Despite some past studies showing an increased sensorimotor GABA concentration correlating with better tactile acuity performance (Puts et al., 2011; Kolasinski et al., 2017), there is also evidence that lower M1 GABA concentration correlates with better motor learning capabilities in a healthy sample (Stagg et al., 2011b). Our results did not show any correlation between sensorimotor GABA concentration and tactile acuity in either healthy control subjects or CRPS patients. This difference with previous studies may result from the fact that we measured tactile acuity using a two-point discrimination test, whereas in previous studies subjects were asked to either discriminate whether two different vibration frequencies were the same or to identify which finger was stimulated first. We found a ceiling effect in the performance of the tactile acuity task in our healthy control subjects however we justify our choice of test; two-point discrimination has been more widely used to measure tactile acuity in CRPS patients than other techniques, and the method of two-point discrimination testing has largely been standardized in pain studies (save for the type of tool; e.g., callipers or wheels; Cashin and McAuley, 2017). Nevertheless, future studies should evaluate the difference in tactile acuity assessment via discrimination and temporal order judgment tasks to understand whether different tactile assessments can lead to different tactile acuity outcomes and relationships with GABA. This is important in a clinical setting, given that tactile acuity training reportedly helps to improve tactile acuity as well as reduce pain intensity in CRPS (David et al., 2015; Schmid et al., 2017).

There are several limitations needing discussion. First, we investigated a limited number of CRPS subjects, although our sample size is considerable given the relatively rare nature of this disorder, and this sample is larger than most previous neuroimaging studies. Given the negative results presented in this study, it is possible that a type 2 error has occurred (i.e., that our sample size is underpowered and that differences may exist with larger numbers). However, there were no signs of trends in the main analyses, suggesting that if there are such differences in total neurotransmitter content they are likely to be small and therefore perhaps unlikely to explain CRPS pathophysiology. Second, as with other neuroimaging studies in CRPS, our investigation had a cross-sectional design. We did not assess individuals before they developed CRPS and so cannot determine whether S1/M1 biochemical changes occur throughout the course of CRPS, for example during the establishment of CRPS, and whether they may then return to control levels in the long term. A larger sample of patients, recruited early in the course of the disorder and followed longitudinally, would ideally shed light on this. Of course it would be ideal to recruit only unilateral or bilateral CRPS patients, rather than both. However, not only would this be challenging to recruit, it would not reflect the reality of the clinical setting. Furthermore, in this study we were still able to demonstrate the difference in TPD between hands across the CRPS group despite mixed presentations. Finally, it is crucial to take into account methodological considerations that may impact on our findings. For instance, measurements of sensorimotor biochemical concentrations are affected by the size of the area investigated, which, because of the low signal-to-noise ratio of GABA, was large (30 × 30 × 30 mm) in this investigation (Mullins et al., 2014). While this relatively large voxel size provides better signal quality, it may encompass brain areas outside S1/M1, and, furthermore, our results may have been different if we were able to restrict our investigation to the area of specifically S1 or M1. In addition, the large voxel size covers areas of different tissues, that is, the voxel includes gray matter, white matter, and CSF, tissues that have different biochemical concentrations. While it is likely that there were differences between subjects with respect to the proportions of different tissue types with the region sampled, we limited such effects on GABA and glutamate concentrations by performing voxel parcellations to confirm that fractions of brain tissue were not different between control subjects and CRPS subjects. Movement is an important methodological consideration with MRS. Ideally, we would have acquired a second anatomical scan after the MRS sequence to determine whether participants had moved in the scan; however, we could not practically achieve this. Finally, it is important to note that the experimenter testing tactile acuity was not blinded to participant grouping, and this inherently poses a risk of inflation of effect size. What we can say is that the administration of this test was delivered consistently across all participants.

As with any clinical disorder, we recruited a group taking a variety of medications. The research suggests that the effect of medications on GABA levels in the brain is specific to brain region and to pathology. There is not an extensive amount of literature on medication effects on GABA in the sensorimotor cortex in chronic pain, as there is for areas such as the occipital cortex in disorders such as epilepsy (Puts and Edden, 2012). Of the drugs most commonly used over the past 24 h in our sample, the two most worthy of comment are gabapentin and pregabalin, both GABA-derivative drugs. These two are known to exert an effect on the GABAergic system and have been found to decrease pain levels. Their mechanism of action is not fully understood, and they are not universally effective (Enna and McCarson, 2006). These drugs were used by less than half of CRPS participants in the last 24 h, and thus we think it unlikely that our negative findings are due to any masking of results by medications.

In conclusion, our findings show that individuals with upper limb CRPS display reduced tactile acuity in the painful hand compared with the nonpainful hand and pain-free control subjects. Despite this acuity difference, CRPS was not associated with altered GABA or glutamate concentrations within the sensorimotor cortex on either the side that represents the affected hand or the side that represents the unaffected hand. While these results appear to be at odds with the sensorimotor cortex disinhibition reported in investigations using TMS, it is possible that GABAergic mechanisms other than total GABA concentration, as measured here with MRS, may contribute to such disinhibition. Clearly, further work with larger samples would confirm these findings. More importantly, future studies should investigate sensorimotor GABA in relation to other GABAergic mechanisms, such as GABA_A_ receptor activity, to determine whether other GABAergic mechanisms can explain disinhibition of CRPS and in turn provide direction for targeted restoration of inhibition.

## Synthesis

Reviewing Editor: Karen Davis, Krembil Research Institute and University of Toronto Decisions are customarily a result of the Reviewing Editor and the peer reviewers coming together and discussing their recommendations until a consensus is reached. When revisions are invited, a fact-based synthesis statement explaining their decision and outlining what is needed to prepare a revision will be listed below. The following reviewer(s) agreed to reveal their identity: Petra Schweinhardt.

In this study, CRPS patients are investigated using proton magnetic resonance spectroscopy (1H-MRS). A MEGA PRESS sequence is used with the aim of measuring GABA concentrations (and glutamate + glutamine = Glx) in the sensorimotor cortex. The rationale for doing so is that the functional measures, using transcranial magnetic stimulation, have repeatedly suggested that CRPS patients show disinhibition in S1/M1. The rational is sound and the study appears well conducted. 14 patients with CRPS are compared to 20 age-matched controls (selected from a larger set of controls), As previous studies have shown, two-point discrimination of the affected hand is impaired in patients. Contrary to the hypothesis, GABA levels in S1/M1 are not different between patients and controls or between the hemispheres corresponding to the (more) affected hand vs. the hemispheres corresponding to the less/un-affected hand.

Overall impression:

MRS studies in pain patients are relatively rare and it is difficult to conduct larger studies in CRPS patients. Thus, this negative finding has value but given the low sample size, it is not adequately powered to make a firm conclusion regarding the null result presented and so the findings need to be discussed in a more balanced way.

Major Comments

1. Confidence in a null result: The negative finding needs to be interpreted with more caution. Throughout the manuscript, it is taken as evidence of absence, i.e. as evidence that tonic GABA levels are indeed not different in CRPS patients in S1/M1. The possibility of a Type 2 error as well more methodological considerations why no differences in GABA levels might have been observed has to be explicitly discussed (and the interpretation of a negative finding toned down throughout the manuscript). Also, a power analysis needs to be performed and included.

2. Power: The author's discussion on line 395-399 regarding statistical power is slightly confusing. They argue that because they were able to find the significant behavioural result, they were adequately powered to find the MRS result: this does not seem correct to me. I understand why the CRPS sample size is low- it is challenging to recruit this kind of patient group. However, I'm not sure that a study with a group size in the low teens can ever confidently be interpreted as the authors do: “the study is not underpowered - just there is no difference in S1/M1 GABA or glutamate concentrations in CRPS subjects compared with controls”.

3. Sample size: Throughout the manuscript, there is confusion w.r.t. the number of controls and patients (45 controls in abstract, 30 in line 96; patients: 14 in results; 15 in discussion:15).

4. The large voxel size is correctly discussed as possibly precluding the detection of a difference in GABA levels between patients and controls. Furthermore, medication (do any of the medications have known effects on GABA levels?) might mask a difference. The MEGA PRESS sequence is suited for measuring GABA (and somewhat Glx) but particularly sensitive to subject movement - was a second anatomical scan acquired after MRS so that movement parameters could be estimated?

5. Plots: I would advise that instead of bar plots, some alternative format that shows the individual data points would be advisable. This is specifically true given the small sample size. For analyses which involve a within-subject comparison bar plots do not clearly allow for the data to be inspected: a plot showing the pairwise connections between datapoints would be very helpful.

6. Age differences between the groups: I appreciate that the authors have adjusted their CRPS vs. control comparisons to include fewer control participants who are best age matched. Given the marked age difference between the original two groups, I think it would be worthwhile plotting the age data on the level of individual data points across groups, and highlighting those control participants who are included in the CRPS comparison. I am concerned that the age range is potentially interacting with the comparisons made e.g. in the 28-participant analysis of dominant vs. non-dominant GABA, there is no significant difference, but in the dominant vs. non-dominant figures for the older control group presented indirectly on lines 237-239, it seems there is a more marked difference in the GABA values. Did the authors perform any correlational analyses to assess whether the GABA values or the interhemispheric differences with GABA were correlated? One key difference between this study and the other studies of GABA vs. tactile acuity would appear to be a markedly older cohort.

7. Tactile acuity: I wonder if their authors could expand on their tactile acuity task. Firstly, it would be useful if the authors could provide goodness of fit measures for their logistic regression; were participants excluded on the basis of a poor fit? Secondly, it seems from the example participant in Figure 1A that participants perform at ceiling on 3 of the 5 trial conditions, was this a common feature of all participants? Thirdly, why were so few trials used for a model fit? I understood from the methods that there were 7 trials per distance- meaning the potential % correct values for each category in each participant are 0,14.3%,28.5%,42.9%,57.1%,71.4%,85.7%,or 100%- yet on the example participant plot, there seem to be data points (grey squares) in the upper 90% range- is this an averaged plot across multiple participants? Also, was the experimenter manually delivering the stimuli blinded to the participant grouping?
